# MPEK: a multitask deep learning framework based on pretrained language models for enzymatic reaction kinetic parameters prediction

**DOI:** 10.1093/bib/bbae387

**Published:** 2024-08-12

**Authors:** Jingjing Wang, Zhijiang Yang, Chang Chen, Ge Yao, Xiukun Wan, Shaoheng Bao, Junjie Ding, Liangliang Wang, Hui Jiang

**Affiliations:** State Key Laboratory of NBC Protection for Civilian, No. 37 South Central Street, Yangfang Town, Changping District, Beijing 102205, China; State Key Laboratory of NBC Protection for Civilian, No. 37 South Central Street, Yangfang Town, Changping District, Beijing 102205, China; State Key Laboratory of NBC Protection for Civilian, No. 37 South Central Street, Yangfang Town, Changping District, Beijing 102205, China; State Key Laboratory of NBC Protection for Civilian, No. 37 South Central Street, Yangfang Town, Changping District, Beijing 102205, China; State Key Laboratory of NBC Protection for Civilian, No. 37 South Central Street, Yangfang Town, Changping District, Beijing 102205, China; State Key Laboratory of NBC Protection for Civilian, No. 37 South Central Street, Yangfang Town, Changping District, Beijing 102205, China; State Key Laboratory of NBC Protection for Civilian, No. 37 South Central Street, Yangfang Town, Changping District, Beijing 102205, China; State Key Laboratory of NBC Protection for Civilian, No. 37 South Central Street, Yangfang Town, Changping District, Beijing 102205, China; State Key Laboratory of NBC Protection for Civilian, No. 37 South Central Street, Yangfang Town, Changping District, Beijing 102205, China

**Keywords:** multitask deep learning, pretraining, enzymatic reaction, *k*cat prediction, *K*m prediction

## Abstract

Enzymatic reaction kinetics are central in analyzing enzymatic reaction mechanisms and target-enzyme optimization, and thus in biomanufacturing and other industries. The enzyme turnover number (*k*_cat_) and Michaelis constant (*K*_m_), key kinetic parameters for measuring enzyme catalytic efficiency, are crucial for analyzing enzymatic reaction mechanisms and the directed evolution of target enzymes. Experimental determination of *k*_cat_ and *K*_m_ is costly in terms of time, labor, and cost. To consider the intrinsic connection between *k*_cat_ and *K*_m_ and further improve the prediction performance_,_ we propose a universal pretrained multitask deep learning model, MPEK, to predict these parameters simultaneously while considering pH, temperature, and organismal information. Through testing on the same *k*_cat_ and *K*_m_ test datasets, MPEK demonstrated superior prediction performance over the previous models. Specifically, MPEK achieved the Pearson coefficient of 0.808 for predicting *k*_cat_, improving ca. 14.6% and 7.6% compared to the DLKcat and UniKP models, and it achieved the Pearson coefficient of 0.777 for predicting *K*_m_, improving ca. 34.9% and 53.3% compared to the Kroll_model and UniKP models. More importantly, MPEK was able to reveal enzyme promiscuity and was sensitive to slight changes in the mutant enzyme sequence. In addition, in three case studies, it was shown that MPEK has the potential for assisted enzyme mining and directed evolution. To facilitate *in silico* evaluation of enzyme catalytic efficiency, we have established a web server implementing this model, which can be accessed at http://mathtc.nscc-tj.cn/mpek.

## Introduction

Enzymatic reactions are widely used in manufacturing pharmaceutical products and chemicals and in generating energy, owing to their high efficiency, environmental safety, and high stereoselectivity [1–3]. In this respect, they are central in developing carbon-neutral industries and are favored over traditional chemical synthesis approaches. In synthetic biology, heterologous biosynthesis based on a biological chassis relies on optimizing and designing biosynthetic routes comprising a series of enzymatic reactions [4]. In this context, enzymes often play an important role like ‘chips’, and the iterative upgrading of these ‘chips’ requires analysis of enzymatic reaction mechanisms and optimization of enzyme-directed evolution.

The study of enzymatic reaction kinetics is crucial for an in-depth understanding of enzymatic reaction mechanisms and efficient target-enzyme optimization [5, 6]. The enzyme turnover number (*k*_cat_, the maximal rate of product formation at a saturating substrate concentration) and Michaelis constant (*K*_m_, reflecting the affinity between the enzyme and the substrate) are the two most important parameters for measuring an enzyme’s catalytic efficiency [7]. However, determining these parameters via biological experimentation is time-, labor-, and cost-intensive [8]. With the accumulation of experimental data and the development of intelligent algorithms, machine learning-based prediction has been developed as a powerful approach for efficiently determining these kinetic parameters [9, 10]. Borger et al. [9] constructed a linear regression model to predict *K*_m_ for eight metabolites under various enzyme–organism reactions, obtaining a root mean square error (RMSE) of 1.01. Yan et al. [10] constructed a feedforward backpropagation neural network model for predicting the *K*_m_ of cellobiose catalyzed by 36 β-glucosidases, obtaining a Pearson coefficient (PCC) > 0.6 on a validation dataset consisting of 12 β-glucosidases.

The continuous development of deep learning with more powerful feature extraction and complex data processing capabilities has advanced research into the prediction of *k*_cat_ and *K*_m_ [11–15]. For example, Heckmann et al. [11] successfully constructed a model that predicts *k*_cat_ in the metabolic network of *Escherichia coli*. This study enhanced the understanding of the kinetome of *E. coli* as well as quantitative proteomics. However, the single enzyme type, small dataset, and complex input features (including metabolic networks, protein structures, and substance concentration) may make it difficult to apply to other species. Kroll et al. [12] used molecular fingerprints, octanol–water partition coefficient, and molecular weights to characterize substrates, and UniRep [16] to characterize 11 675 wild-type enzymes. They constructed a gradient-boosting regression model to predict *K*_m_, obtaining the *R*^2^ of 0.53 on a test dataset of 2335 enzymes (hereafter Kroll_model). Li et al. [13] proposed a DLKcat model based on 16 838 *k*_cat_ entries, which used graph neural network (GNN) and convolutional neural network (CNN) to extract the substrate’s molecular graph information and enzyme’s sequence information, respectively, and used a neural attention mechanism to predict *k*_cat_. DLKcat showed superior prediction performance in the test dataset of 1684 entries (PCC = 0.71). Kroll et al. [14] developed TurNup to predict the *k*_cat_ values of wild-type enzymes. The model utilized data from 4271 wild-type enzymes, and utilized a modified ESM-1b pretrained language model to represent the enzymes and differential reaction fingerprints to represent the complete enzymatic reaction. A gradient-boosting algorithm was used to predict *k*_cat_, achieving *R*^2^ = 0.44 and PCC = 0.67 on a test dataset of 850 entries. Subsequently, Yu et al. [15] proposed UniKP, a two-layer framework based on a pretrained language model, achieving robust prediction of *k*_cat_ while considering enzymatic reaction pH and temperature. UniKP achieved the optimal performance on a test dataset of 1684 entries with an *R*^2^ of 0.68. UniKP has been extended to *K*_m_ and *k*_cat_/*K*_m_ datasets, achieving superior performance.

These studies have demonstrated the advantage of deep-learning techniques over traditional machine-learning methods in predicting enzymatic reaction kinetic parameters. However, the current predictive models still have much room for improvement due to the limitations of the small number of datasets, insufficient types of enzymes, and incomplete structural characterization. Based on the ongoing development of pretrained models for proteins and small molecules [17–21], molecular characterization using large-scale pretrained models is now feasible, and such models effectively compensate for insufficient molecular characterization and small sample sizes [22–24].

More importantly, considering that *k*_cat_ and *K*_m_ are intrinsically connected [15, 25], and there is no unified model that can learn this intrinsic connection, it is highly judicious to apply multitask learning to learn this connection and predict *k*_cat_ and *K*_m_ simultaneously. Because multitask deep learning is a joint learning paradigm that learns the intrinsic correlation between multiple related tasks and leads to the simultaneous prediction of multiple tasks, it would benefit for improving predictive performance, reducing the overall computational cost, and enhancing generalizability [26]. It has attracted wide attention in areas such as health informatics, computer vision, natural language processing, and so on [27–31].

Therefore, we propose a universal multitask deep learning framework based on pretrained language models, named MPEK, to achieve predicting *k*_cat_ and *K*_m_ simultaneously. Moreover, considering that enzymatic reaction kinetic parameters are significantly influenced by environmental factors such as pH and temperature [32, 33], MPEK considers these factors as well as organismal information, allowing comprehensive description of enzymatic reaction and effective prediction of *k*_cat_ and *K*_m_ simultaneously. Independent test results show that MPEK can reveal enzyme promiscuity and is sensitive to slight changes in mutant enzyme sequences. Moreover, it outperforms other state-of-the-art models on the same test dataset. In addition, MPEK has the potential to assist for enzyme mining and directed evolution. Collectively, MPEK provides important theoretical support for enzyme mining and optimization and the mechanistic analysis of enzymatic reactions, thus accelerating the discovery of novel target enzymes.

## Results

### MPEK framework

**Figure 1:**
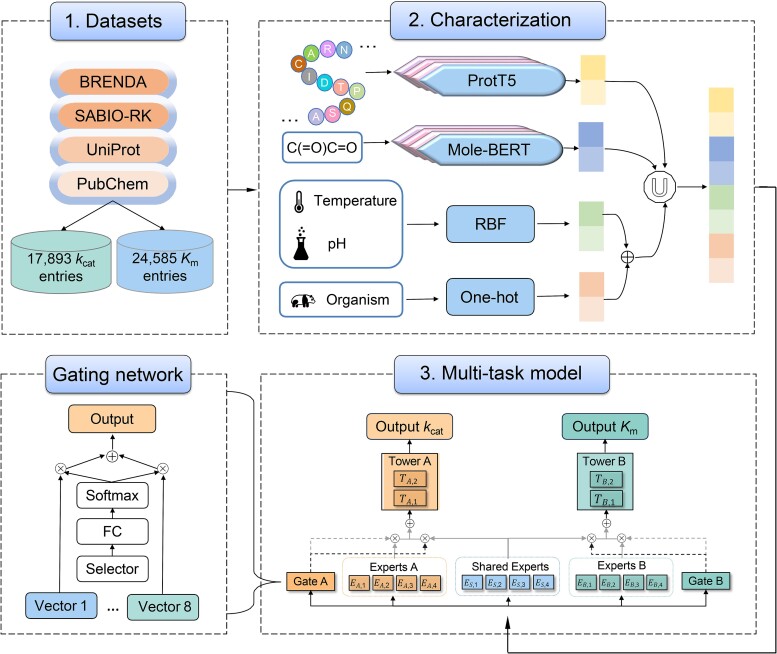
Overview of the MPEK framework. Part 1 is the dataset collection and processing of MPEK. The data are obtained from the BRENDA and SABIO-RK databases, the amino acid sequences of the enzymes and the SMILES of the substrates are collected from UniProt and PubChem databases, respectively. 17 893 kcat entries and 24 585 *K*_m_ entries are collected after several rounds of processing. Part 2 is the feature characterization. ProtT5 and Mole-BERT large-scale language models are used to encode enzyme and substrate features, respectively. pH, temperature, and organisms are encoded using RBF and one-hot, respectively, and the encoded feature vectors are summed and contacted with those of the enzymes and substrates. Part 3 is the multitask learning architecture. The characterized enzymatic reaction feature vectors are inputted into the CGC multitask deep learning model, and the contribution of shared and specific information between *k*_cat_ and *K*_m_ to each prediction task is dynamically adjusted through the gating network, and then the simultaneous prediction of *k*_cat_ and *K*_m_ is achieved.

The MPEK model (Fig. 1) is based on the customized gate control (CGC) framework for predicting *k*_cat_ and *K*_m_. The pipeline comprises three components: dataset preparation, characterization, and a multitask model. The datasets were collected from the BRENDA [34], SABIO-RK [35], UniProt [36], and PubChem [37] databases. ProtT5-XL-U50 (hereafter ProtT5) [17] and Mole-BERT [20], pretrained language models, were used to characterize the enzymes and substrates, respectively. One-hot encoding [38, 39] was used to encode the organism, and radial basis function (RBF) [40, 41] was used to encode pH and temperature. The extracted feature vectors were summed and then concatenated with those of enzymes and substrates for use as inputs to the downstream CGC multitask model. To reduce the negative transfer and the seesaw phenomena in multitask prediction [42–44], the shared module and the two expert modules (A and B) were used to predict *k*_cat_ and *K*_m_, and the feature vectors from the expert modules were dynamically fused with those from the shared module via a gating network. The fused feature vectors were used as inputs for each tower network, thereby predicting *k*_cat_ and *K*_m_ accurately and simultaneously.

### Statistical analyses of datasets

To fully evaluate the type and quality of the collected data, the dataset was analyzed in detail (Fig. S1). The values of *k*_cat_ and *K*_m_ generally conformed to a normal distribution (Fig. S1a, b). Seven enzymes were included, with oxidative hydrolases contributing the most entries (10 644), accounting for 37.69% of all entries (Fig. S1c). The whole data contains 1460 organisms, of which *Homo sapiens* data comprises the largest component, with 2671 and 1179 entries for wild-type and mutant enzymes, respectively (Fig. S1d). To ensure model validation reliability, the training, validation, and test datasets were randomly split to contain entries with only *k*_cat_ entries, only *K*_m_ entries, and both, according to the missing status of *k*_cat_ and *K*_m_ labels, and the proportions of the three types of entries were almost the same (Fig. S2).

### Performance comparison with other methods

**Figure 2:**
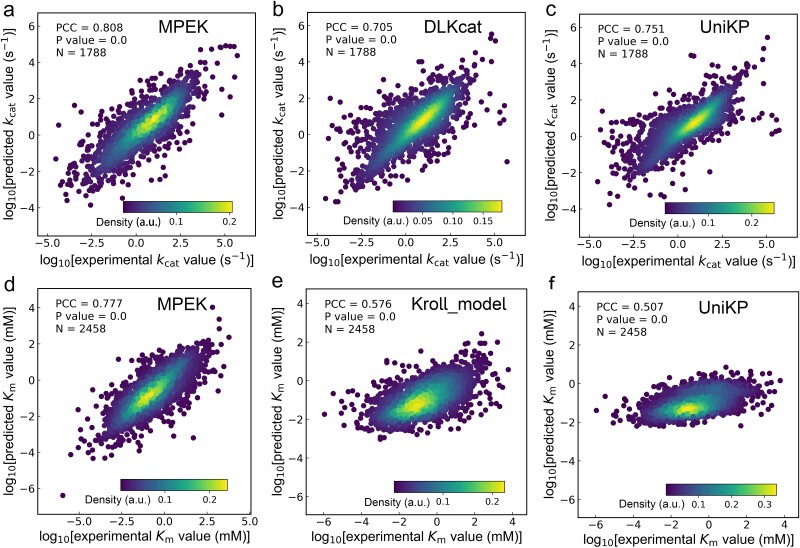
Performance comparison of MPEK with other excellent models. a-c: Independent test performance of MPEK, DLKcat, and UniKP on the *k*_cat_ test dataset, respectively. d-f: Independent test performance of MPEK, Kroll_model, and UniKP on the *K*_m_ test dataset, respectively. The brightness of color represents the density of data points. Student’s *t*-test was used to calculate the *P* value for PCC. *N* represents the number of entries in the test dataset. Source data are provided as a source data file.

The MPEK was trained and tested on a dataset containing 17 893 *k*_cat_ entries and 24 585 *K*_m_ entries. The datasets were randomly divided into training, validation, and test datasets in a ratio of 8:1:1. The training and validation datasets were used for model tuning, while the test dataset was used for independent testing of the trained models. To evaluate the predictive performance of MPEK, we compared it with three excellent *k*_cat_ or *K*_m_ prediction models. Since TurNup additionally requires information on the products of enzymatic reaction, it will not be comparable with MPEK. Therefore, DLKcat, UniKP, and Kroll_model were selected for comparison, where DLKcat and UniKP implement *k*_cat_ prediction, which can be used to compare the *k*_cat_ prediction task, and UniKP and Kroll_model implement the *K*_m_ prediction, which can be used to compare the *K*_m_ prediction task. We tested the saved MPEK, DLKcat, and UniKP models on the test dataset of 1788 *k*_cat_ entries, and tested the saved MPEK, Kroll_model, and UniKP models on the test dataset of 2458 *K*_m_ entries. When predicting *k*_cat_ using the *k*_cat_ test dataset, PCCs of 0.808, 0.705, and 0.751, respectively, were obtained for MPEK, DLKcat, and UniKP, and MPEK improved prediction performance by ca. 14.6% and 7.6%, respectively, over that of DLKcat and UniKP (Fig. 2a, b, c). When predicting *K*_m_, PCCs of 0.777, 0.576, and 0.507, respectively, were obtained for MPEK, Kroll_model, and UniKP, and MPEK improved prediction performance by ca. 34.9% and 53.3%, respectively, over that of Kroll_model and UniKP (Fig. 2d, e, f). Therefore, MPEK performed best for the test datasets for both tasks, primarily because it was trained with much more data than the previously published models. Furthermore, MPEK not only uses large pretrained language models to extract enzyme–substrate features, but also considers auxiliary (pH, temperature, and organismal) information to more comprehensively characterize enzymatic reactions.

### MPEK performance with different pretrained language models

**Figure 3:**
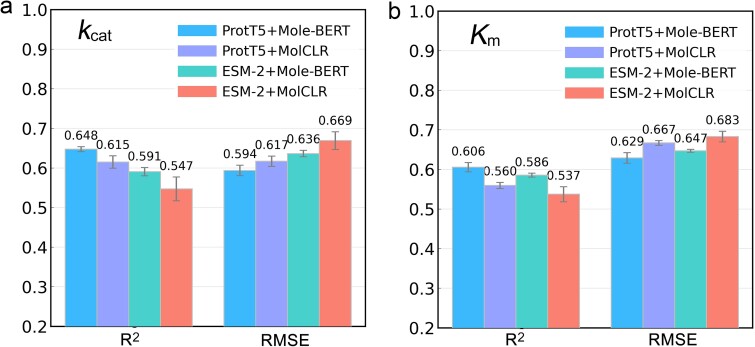
Performance comparison of different feature extraction combinations on test datasets. This is an average result by randomly splitting the training, validation, and test datasets five times. Panels a and b are for the *k*_cat_ and *K*_m_ prediction tasks, respectively. Source data are provided as a source data file.

We experimentally compared two frequently used protein pretrained models, ProtT5 [17] and ESM-2 [18], to extract enzyme sequence features, and two small-molecule pretrained models, MolCLR [19], and Mole-BERT [20], to extract substrate structural features. Four feature-extraction combinations were generated: ProtT5 + Mole-BERT, ProtT5 + MolCLR, ESM-2 + Mole-BERT, and ESM-2 + MolCLR. We constructed models using each of the four feature-extraction combinations and tested them after tuning hyperparameters to compare their predictive performances for *k*_cat_ and *K*_m_. The ProtT5 + Mole-BERT combination exhibited the best model-testing performance, achieving average *R*^2^ and RMSE values of 0.648 and 0.594, respectively, for *k*_cat_ (Fig. 3a), and of 0.606 and 0.629, respectively, for *K*_m_ (Fig. 3b). For *k*_cat_, ProtT5 + MolCLR performed better than ESM-2 + Mole-BERT and ESM-2 + MolCLR, whereas for *K*_m_, ESM-2 + Mole-BERT performed better than ProtT5 + MolCLR and ESM-2 + MolCLR. ProtT5 and Mole-BERT are therefore more appropriate than ESM-2 and MolCLR for extracting enzyme–substrate features to predict enzymatic reaction kinetic constants.

MPEK additionally encodes auxiliary information, including information about the enzymatic reaction environment (pH and temperature) and the organism. Including this information improved *k*_cat_ and *K*_m_ prediction (Fig. S3), consistent with the results that adding pH or temperature information to UniKP improves predictive performance [15]. Auxiliary information can thus be incorporated when predicting enzyme kinetic parameters.

### MPEK performance using different downstream multitask models

A rational multitask learning architecture will lead to superior performance. To evaluate the impact of multitask learning architecture on model performance, we tested two classic soft-parameter-sharing multitask learning frameworks, CGC and progressive layered extraction (PLE) [45]. PLE extends the single-layer CGC structure to multiple layers, enabling learning of semantic representations of increasing depth. The CGC-based model performed significantly better than the PLE-based model. For *K*_m_, using the test datasets, CGC achieved an average *R*^2^ that was 10.2% higher than that achieved by PLE (Fig. S4). This indicates that the CGC multitask learning framework, which has fewer expert layers and simpler models, is more suitable for predicting enzymatic reaction kinetic parameters.

### Multitask learning improves MPEK performance

MPEK employs CGC-based multitask learning to predict *k*_cat_ and *K*_m_ efficiently and simultaneously. To verify the rationality of employing multitask learning, we compared the simultaneous multitask and single-task prediction models for *k*_cat_ and *K*_m_. Multitask modeling improved *k*_cat_ and *K*_m_ prediction, achieving improvements in *R*^2^ of 2.4% and 4.5%, respectively (Fig. S5). This suggests that multitask deep learning may capture the intrinsic connection between *k*_cat_ and *K*_m_ and improve predictive performance by adding shared information.

### MPEK performance for wild-type and mutant enzymes

To test the ability of MPEK to predict *k*_cat_ and *K*_m_ for wild-type and mutant enzymes, we divided the test dataset into wild-type and mutant enzyme subtest datasets based on the original annotation information. There were 1143 and 645 wild-type and mutant enzymes, respectively, in the *k*_cat_ subtest dataset, and 1690 and 768, respectively, in the *K*_m_ subtest dataset. Independent tests were performed on each of the two subtest datasets. Using the wild-type and mutant subtest datasets achieved PCCs of 0.786 and 0.831, respectively, for *k*_cat_ (Fig. S6a, b) and 0.750 and 0.835, respectively, for *K*_m_ (Fig. S6c, d). Based on these findings, MPEK exhibits good predictive ability for both the wild-type and mutant enzymes.

**Figure 4:**
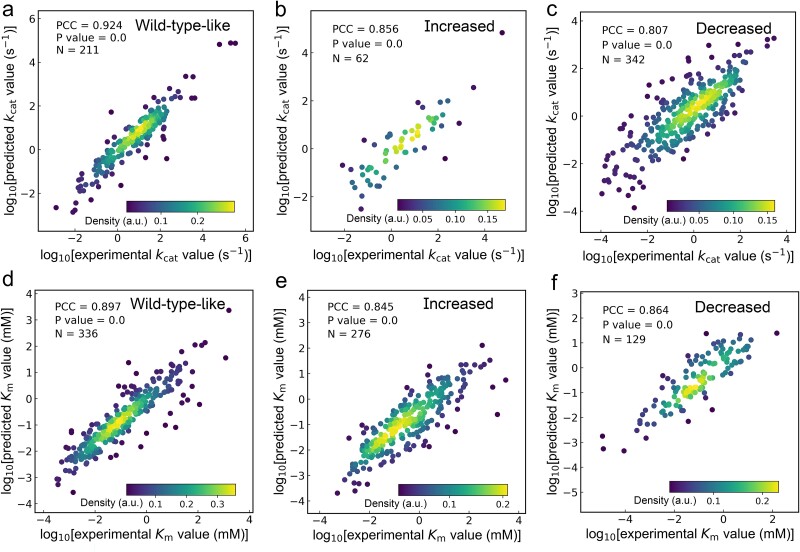
Performance of MPEK on wild-type-like, increased, and decreased subtest datasets. a-c: PCCs of the three subtest datasets divided from the *k*_cat_ test dataset. d-f: PCCs of the three subtest datasets divided from the *K*_m_ test dataset. The brightness of color represents the density of data points. Student’s *t*-test was used to calculate the *P* value for PCC. *N* represents the number of entries in each subtest dataset. Source data are provided as a source data file.

MPEK captured the effects of slight changes in enzyme sequences for *k*_cat_ and *K*_m_ prediction. We further split the mutant subtest dataset into three categories: wild-type-like, increased, and decreased. For the *k*_cat_ mutants, the wild-type-like, increased, and decreased categories exhibited PCCs of 0.924, 0.856, and 0.807, respectively (Fig. 4a, b, c), for *K*_m_, they exhibited PCCs of 0.897, 0.845, and 0.864, respectively (Fig. 4d, e, f), indicating good test performance.

### MPEK can reveal enzyme promiscuity

**Figure 5:**
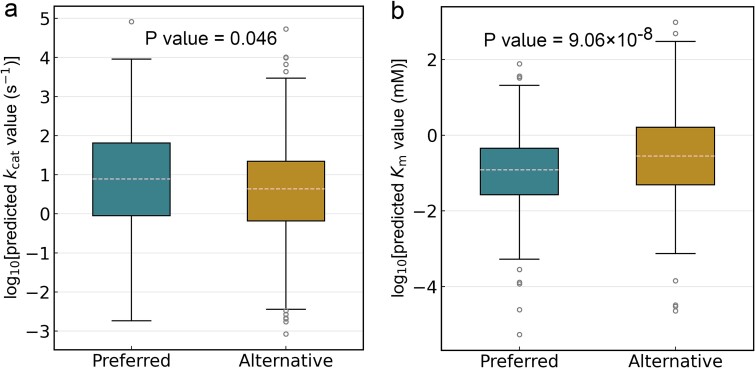
The prediction of enzyme promiscuity. Panels a and b are enzyme’s promiscuity prediction for *k*_cat_ and *K*_m_ test datasets, respectively. A two-sided Wilcoxon rank sum test was used to calculate the *P* values. Source data are provided as a source data file.

Understanding enzyme promiscuity is a key topic in evolutionary biology [46, 47]. MPEK was able to reveal enzyme promiscuity. For enzymes with multiple substrates, we divided promiscuous substrates in the test dataset into two categories based on their experimentally measured *k*_cat_ and *K*_m_ values: preferred substrates (*k*_cat_: 178 entries; *K*_m_: 277 entries) and alternative substrates (*k*_cat_: 519 entries; *K*_m_: 844 entries). The detailed data classification can be found in 4.1.1. The MPEK-predicted log_10_-transformed *k*_cat_ values were higher for the preferred substrates (median *k*_cat_ = 0.891 s^−1^) than for the alternative substrates (median *k*_cat_ = 0.638 s^−1^; Fig. 5a, *P* < 0.05). Similarly, for the *K*_m_ test dataset, the MPEK-predicted log10-transformed *K*_m_ values were lower for the preferred substrates (median *K*_m_ = −0.916 mM) than for the alternative substrates (median *K*_m_ = −0.549 mM; Fig. 5b, *P* < 0.05), which is consistent with the principle that smaller *K*_m_ has a higher binding affinity.

### MPEK performance for unseen enzymes or substrates

Predicting enzyme kinetic parameters for unseen entities allows researchers to explore novel enzymes or substrates. This is particularly valuable for mining new enzymes, enzyme-directed evolution and enzyme-substrate interaction studies. To evaluate the performance of MPEK in predicting novel enzymatic reactions, strict subtest datasets, excluding either the enzyme sequences or substrates included in the training dataset, were created from the test dataset. The strict *k*_cat_ and *K*_m_ subtest datasets comprised 398 and 544 entries, respectively. Based on independent tests, PCC reached 0.775 for the *k*_cat_ and 0.735 for the *K*_m_ (Fig. S7a, b). In addition, based on the enzyme’s sequence identity between the test dataset and the training dataset, the cd-hit-2d [48] was used to divide the test dataset into six subtest datasets. After independent testing, it was found that when the sequence identity is lower than 40%, MPEK has poor prediction ability for *k*_cat_, but still has some prediction ability for *K*_m_ (Fig. S8). This indicates that the data volume and diversity of the model need to be further improved. Currently, when the sequence identity of our training set is greater than 40%, MPEK will have some predictive potential for unknown enzymatic reactions and can be used to predict the enzyme kinetic constants of the enzymatic reaction under evaluation.

### MPEK assistance in enzyme mining and evolution

The identification of substitute enzymes with enhanced activity for specific biochemical reactions, and improving the efficiency of known enzymes through directed evolution, are pivotal objectives in synthetic biology and biochemistry research. However, the process of enzyme mining and evolution is cost, time, and labor intensive [25, 49]. For example, Jendresen et al. [49]. identified 107 representative tyrosine ammonia lyases (TALs) from 4729 unique sequences found in more than ten distinct microbial species using bioinformatic analysis. Nevertheless, enzymatic assays indicated that only four TALs displayed increased activity. Chen et al. [25] developed a biosensor for the directed evolution of myrcene synthase, which screened 100 mutants out of 218 random mutagenesis for experimental validation. The results found that only eight myrcene synthase mutants had increased β-myrcene production.

**Figure 6:**
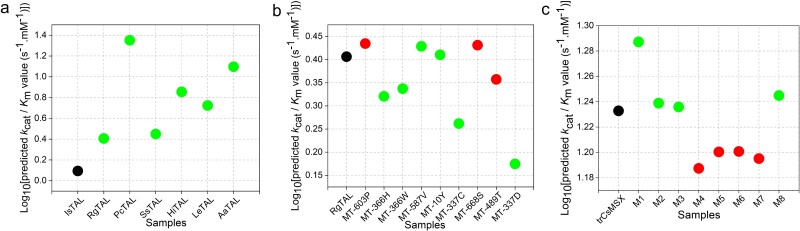
MPEK has potential for enzyme mining and evolution. a-c: MPEK predicted *k*_cat_/*K*_m_ values on tyrosine ammonia lyase (TAL)-homologue dataset, TAL-directed evolution dataset, myrcene synthases directed evolution dataset, respectively. The black, green, and red dots indicate the samples used as reference, the correctly predicted samples, and the incorrectly predicted samples, respectively. IsTAL: TAL from Ilyonectria sp. MPI-CAGE-AT-0026 (KAH6995648.1). RgTAL: TAL from *Rhodotorula glutinis* (AGZ04575.1). PcTAL: TALfrom *Puccinia coronata* f. sp. avenae (PLW06342.1). SsTAL: TAL from *Sporidiobolus salmonicolor* (CEQ38810.1). HiTAL: TAL from *Heterobasidion irregulare* TC 32–1 (XP_009553370.1). LeTAL: TAL from *Lentinula edodes* (KAF8828722.1). AaTAL: TAL from *Aspergillus arachidicola* (KAE8337485.1). M1–M8 are different mutants 25. Source data are provided as a source data file.

To validate the ability of MPEK-assisted enzyme mining and directed evolution, we collected three case study datasets based on published articles with three criteria: (i) The literature was published after 2023 to reduce the possibility of case study datasets appearing in the training dataset, (ii) the enzyme-directed evolution and enzyme mining datasets containing *k*_cat_ and *K*_m_ values; and (iii) each case study dataset should contain more than six entries. The TAL-homologue data was obtained from the UniKP [15], a dataset containing seven samples that could find sequences from UniProt [36] and had *k*_cat_/*K*_m_ values present. There was no duplication with our training set. Taking the lowest catalytic efficiency, TAL from Ilyonectria sp (IsTAL), as a reference, the catalytic efficiencies of the other six TALs are increased. Independent testing showed that the predicted *k*_cat_/*K*_m_ (the ratio of predicted *k*_cat_ to *K*_m_ values) values were the lowest for IsTAL, and higher for the other six TALs, achieving a hit ratio (HR) of 100% (Fig. 6a).

The TAL-directed evolution dataset was obtained from the UniKP [15] containing ten samples. There was no duplication with our training dataset. Taking wild-type tyrosine ammonia lyase, RgTAL, as a reference, the *k*_cat_/*K*_m_ values are increased for mutants MT-587, MT-10Y, and MT-689 T, and decreased for the remaining five mutations. The independent test showed that the *k*_cat_/*K*_m_ values were increased for mutants MT-587, MT-10Y, MT-366H, and MT-668S, and decreased for the remaining four mutations. MPEK predicted the changing trends in catalytic efficiency for six of the nine mutants, resulting in an HR of ca. 66.7% (Fig. 6b).

The myrcene synthases-directed evolution dataset is derived from our group’s study [25] containing nine samples. The sequence identity with our training dataset is less than 0.52, which was calculated by cd-hit-2d [48]. The myrcene synthase, trCsMSX, had the lowest myrcene production, while the other eight mutant enzymes showed higher myrcene production. Theoretically, the catalytic efficiencies of eight mutants are higher than trCsMSX [25]. Independent testing showed that the predicted *k*_cat_/*K*_m_ values increased for four mutant myrcene synthases, and decreased for the remaining four mutants, resulting in a HR of 50% (Fig. 6c).

Based on the above three case studies, it was revealed that MPEK has potential in guiding enzyme mining and directed evolution. Moreover, MPEK predicted different *k*_cat_ and *K*_m_ values for the directed evolution samples, indicating MPEK can capture the slight sequence changes well. In addition, we tested MPEK server and the models saved by UniKP, DLKcat, and Kroll_model on the TAL-homologue and TAL-directed evolution datasets. These two datasets are also not present in the UniKP, DLKcat, and Kroll_model datasets. We did not quantitatively test the myrcene synthases-directed evolution dataset due to the unavailability of quantitative *k*_cat_ and *K*_m_ data. The test results showed that MPEK had the lowest RMSE for predicting *k*_cat_ compared to these excellent models, while a higher RMSE than UniKP or Kroll_model for predicting *K*_m_ (Table S1). This suggests that MPEK would have a higher reference value for *k*_cat_ than *K*_m_ in guiding enzyme-directed evolution. Overall, based on MPEK-predicted *k*_cat_ and *K*_m_ values, it is possible to screen for efficient effective enzymes, which would greatly improve the efficiency of mining and direct evolution of effective enzymes.

### MPEK web server to facilitate *k*_cat_ and *K*_m_ prediction

We present an online server, at http://mathtc.nscc-tj.cn/mpek, to facilitate the use of MPEK without requiring programming skills or specialized software. This server requires the amino acid sequence of the enzyme and the substrate’s simplified molecular-input line-entry system (SMILES) string as inputs, with organisms, pH, and temperature as optional inputs. It enables the prediction of individual sample by directly inputting information, including the enzyme’s amino acid sequence, the SMILES string of the substrate, pH, and temperature, and organismal information. Furthermore, batch prediction can be achieved by uploading CSV files of up to 1000 entries, substantially improving the efficiency of target-enzyme screening.

## Discussion

Although comprehensive evaluation of an enzyme’s catalytic efficiency is required in biomanufacturing, the technology to achieve this has been lacking. MPEK, our multitask deep-learning model based on pretrained language models, addresses this need, and improves *k*_cat_ and *K*_m_ predictive performance. MPEK, based on 17 893 *k*_cat_ entries and 24 585 *K*_m_ entries from the BRENDA and SABIO-RK databases, uses large pretrained language models such as ProtT5 and Mole-BERT to encode enzyme and substrate molecular features, the RBF to encode pH and temperature information, and one-hot encoding to encode organismal information. The encoded feature vectors are concatenated and fed into the downstream multitask model, simultaneously and accurately predicting *k*_cat_ and *K*_m_ by dynamically fusing the common features of *k*_cat_ and *K*_m_ with features specific to both via a gating network. Using the online MPEK server, users can predict *k*_cat_ and *K*_m_ by simply entering the amino acid sequence of the enzyme and the SMILES string of the substrate; it handles individual sample as well as batch prediction of up to 1000 entries. The code and data for this study have been deposited at https://github.com/kotori-y/mpek, to ensure that the results are reproducible and the work is available.

MPEK achieved superior predictive performance for the overall test dataset (with PCCs of 0.808 for *k*_cat_ and 0.777 for *K*_m_) and for both wild-type enzymes and mutants with slightly altered sequences. In comparison with three other excellent methods for *k*_cat_ or *K*_m_ prediction, MPEK exhibited the best predictive performance. For enzymes with multiple substrates, MPEK was able to show enzyme promiscuity. Testing using strict datasets with enzyme sequences or substrate SMILES excluded from the training dataset revealed that MPEK can predict novel enzymatic reactions. Three case studies demonstrated that MPEK would assist in enzyme mining and directed evolution. MPEK is therefore comprehensive and useful for predicting *k*_cat_ and *K*_m_. Its superior performance results from (i) its comprehensive data collection and rigorous data processing procedures, (ii) the use of large pretrained language models to extract enzyme–substrate features and inclusion of auxiliary information on enzyme reactions to characterize molecules more comprehensively, and (iii) its inclusion of intrinsic connections between *k*_cat_ and *K*_m_ via CGC multitask learning, thus incorporating shared information. The feature space was enhanced to a certain extent to compensate for the amount of data.

Nonetheless, there is room for improvement in the model. For example, although MPEK uses large pretrained language models to extract enzyme sequence information and substrate molecular graph information, it does not directly characterize enzyme–substrate complex structures. To address these gaps, we aim to develop methods to employ experimental or predicted structural data for enzyme–substrate complexes to extract key pocket-related information. In addition, MPEK has not good prediction performance for entries with low-sequence identity between the test and training datasets, and it would be more suitable for *in vitro* kinetic parameters of prediction enzymatic reactions, because it is trained based on *in vitro k*_cat_ and *K*_m_ data. In the future, as the quantity and quality of data available in the enzymology database grows, it is expected to improve the kinetic parameters prediction of enzymatic reactions. Moreover, it would also be expected to improve the accuracy of our screening for potential substrates of the specific enzyme.

MPEK is a universal enzymatic reaction prediction tool that predicts the *k*_cat_ and *K*_m_ efficiently and simultaneously. This model will help to improve the evaluation of enzymatic efficiency and provide important theoretical support for the screening of enzymes and the mechanistic analysis of enzymatic reactions, thereby accelerating the discovery and optimization of novel target enzymes. MPEK is therefore a promising and powerful tool for advancing biocatalysis, drug discovery, metabolic engineering, and other enzyme-dependent catalytic processes.

## Materials and Methods

### Dataset preparation for MPEK development

BRENDA [34] and SABIO-RK [35] databases are two authoritative enzymatic databases. Among them, BRENDA is a comprehensive enzyme information database with abundant enzyme-related information, including enzyme classification, functional properties, catalytic mechanism, etc. SABIO-RK is a database for biological reactions, including kinetic parameters, catalytic mechanisms, enzymes, and substrates involved in the reactions. We downloaded *k*_cat_ and *K*_m_ values along with their enzyme commission (EC) number, enzyme type (wild-type and mutant), organismal information, substrate name, UniProt ID, assay conditions (pH and temperature), *k*_cat_ units, and *K*_m_ units from BRENDA [34] and SABIO-RK [35] on 19 March 2023. Protein sequences were queried using two methods: amino acid sequences were downloaded via UniProt using Biopython (https://biopython.org/) if the UniProt ID was available; otherwise, amino acid sequences were downloaded from BRENDA using the organism’s name and the enzyme’s EC number. Wild-type enzyme sequences were directly mapped, while mutated enzyme sequences were altered according to the mutation sites. The SMILES string of the substrate was extracted to represent its structure by searching the PubChem [37] compound database using the substrate name.

To ensure data quality, several rounds of data cleaning were performed. Entries meeting the following criteria were removed: (i) incomplete entries (any missing for EC number, enzyme type, enzyme sequence, substrate SMILES, pH, temperature, organism, *k*_cat_ value (*K*_m_ value), or *k*_cat_ unit (*K*_m_ unit)); (ii) all duplicates (i.e. entries with identical SMILES strings, amino acid sequences, assay conditions, and organisms); (iii) entries with >128 and ≤ 2 heavy atoms in the substrate; and (iv) entries with substrate SMILES containing “.” or *k*_cat_ or *K*_m_ values ≤0. Specific steps and detailed numbers for the data cleaning can be found in Fig. S9. If there were multiple *k*_cat_ or *K*_m_ values for one substrate and one amino acid sequence at the same pH and temperature, we used the maximum *k*_cat_ and minimum *K*_m_. Substrates typically have synonyms in different databases. Therefore, to filter out redundant entries, we converted substrates into classical SMILES strings to further clean the data.

The final dataset comprised 17 893 unique *k*_cat_ entries and 24 585 unique *K*_m_ entries, which can be combined 28 241 unique entries for multitask learning inputs, including 14 237 entries containing both *k*_cat_ and *K*_m_ values, 3656 containing only *k*_cat_ values, and 10 348 containing only *K*_m_ values. To balance the data distribution of the training, validation, and test datasets, the 14 237, 3656, and 10 348 entries were randomly divided into three datasets at a ratio of 8:1:1, respectively. These split datasets were then summed according to the same proportions so that the final training, validation, and test datasets accounted for 80%, 10%, and 10% of the entire dataset, respectively. The MPEK model exhibited robustness following five times random splits (Figs S10 and S11).

#### Subtest dataset classification

To test MPEK comprehensively, we divided the test dataset into four subtest datasets.

(i) Based on enzyme type, the test dataset was divided into wild-type enzymes (*k*_cat_: 1143 entries; *K*_m_: 1690 entries) and mutant enzymes (*k*_cat_: 645 entries; *K*_m_: 768 entries).
(ii) Based on the experimentally measured maximum *k*_cat_ or minimum *K*_m_, promiscuous wild-type enzymes were classified as preferred substrates (*k*_cat_: 178 entries; *K*_m_: 277 entries) and alternative substrates (*k*_cat_: 519 entries; *K*_m_: 844 entries). Specifically, among the wild-type enzymes in the test dataset, an enzyme is classified as promiscuous if it catalyzes multiple substrates, with one having the highest *k*_cat_ or the lowest *K*_m_ being considered as preferred substrates, while the rest are considered alternative substrates.
(iii) Based on the mutation-induced changes in *k*_cat_ and *K*_m_ (relative to the values for the wild-type), the mutant enzymes were classified into three categories: wild-type-like (0.5–2.0-fold change; *k*_cat:_ 211 entries; *K*_m_: 336 entries); increased (> 2.0-fold change; *k*_cat_: 62 entries; *K*_m_: 276 entries), and decreased (< 0.5-fold change; *k*_cat_: 342 entries; *K*_m_: 129 entries).
(iv) Strict subtest datasets were obtained by excluding either the enzyme sequences or substrates included in the training dataset (*k*_cat_: 398 entries; *K*_m_: 544 entries).

### MPEK input calculation

To characterize the enzyme–substrate complexes, we used two frequent protein pretrained models, ProtT5 [17] and ESM-2 [18], to encode the enzyme sequences, and used two popular small-molecule pretrained models, MolCLR [19] and Mole-BERT [20], to encode the substrates. The two feature vectors were further concatenated using four combinations of these models: ProtT5 + Mole-BERT, ProtT5 + MolCLR, ESM-2 + Mole-BERT, and ESM-2 + MolCLR. The ProtT5 + Mole-BERT combination achieved the best predictive performance. We therefore used ProtT5 and Mole-BERT to encode enzymes and substrates, respectively. Radial basis functions and one-hot coding were used to encode the auxiliary (pH, temperature, and organismal) information.

#### Enzyme encoding

ProtT5 [17] was used to encode enzyme sequences. ProtT5, a self-supervised autoencoder based on a transformer containing 24 layers and 3B parameters, can characterize rich information in the absence of multiple sequence alignment (MSA) or evolutionary information, outperforming other outstanding methods for predicting protein secondary structure. Using ProtT5, we extracted a 1024-dimensional sequence embedding for each residue in the enzyme sequence.

The specific encoding process is as follows: (i) Each amino acid in the sequence is tokenized, position-encoded, and encoded as a fixed-length initial vector. (ii) The initial amino acid vectors are input into the 24 transformer encoder blocks. If it is the first block, the input vector is the initial amino acid vector; otherwise, the input vector is the output vector of the last block. (iii) The output vectors of all of the amino acids ${\boldsymbol{v}}^{\mathrm{n}\times \mathrm{m}}$ (where $n$ is the amino acid length and $m$ is 1024-dimensional hidden vector length) from the last block are mean-pooled to obtain ${\boldsymbol{v}}^{1\times 1024}$, the vector characterizing each enzyme.

#### Substrate encoding

Substrate encoding was performed using Mole-BERT, a context-aware tokenizer for molecular graphs to alleviate negative transfer in GNN training. The training process of the encoder in Mole-BERT is as follows: (i) First, the GNN is used to embed the atoms $\mathcal{V}=\left\{{v}_1,{v}_2,\dots, {v}_n\right\}$ in the molecular graph $\mathcal{G}$ to obtain the hidden vector $\boldsymbol{h}=\left\{{h}_1,{h}_2,\dots, {h}_n\right\}$. (ii) For the *i*-th atom, finding the embeddings $\left\{{e}_1,{e}_2,\dots, {e}_{\left|\mathcal{A}\right|}\right\}$ ($\left|\mathcal{A}\right|=512$) of atom ${h}_i$ in the codebook via vector quantization (VQ) and tokenizing it as ${z}_i$:


(1) \begin{equation*} {z}_{\mathrm{i}}=\mathrm{argmi}{\mathrm{n}}_{\mathrm{j}}{\left\Vert{h}_i-{e}_j\right\Vert}_2 \end{equation*}

(iii) The vector $\boldsymbol{z}=\left\{{z}_1,{z}_2,\dots, {z}_n\right\}$ obtained via tokenization of molecular graph $\mathcal{G}$ is embedded to obtain the vector $\boldsymbol{e}=\left\{{e}_{z_1},{e}_{z_2},\dots, {e}_{z_n}\right\}$, and the loss between $\boldsymbol{h}$ and $\boldsymbol{e}$ is calculated. Via continuous iteration, ${h}_i$ acquires more contextual information and more detailed atomic information than other GNNs.

We used Mole-BERT to characterize the substrate, using $\boldsymbol{h}$ as a representation of the atom $\mathcal{V}$. Mean pooling of $\boldsymbol{h}$ was performed to obtain each molecule’s representation ${\boldsymbol{h}}_{\mathrm{s}} \mid{\boldsymbol{h}}_{\mathrm{s}}\in{\mathbb{R}}^{\mathrm{n}\times 300}$ (where *n* is the number of input entries). Each substrate molecule was mapped onto a 300-dimensional vector.

#### Auxiliary information encoding

We included auxiliary information, including organismal, experimental pH, and temperature information. One-hot encoding [40, 41] was used to encode the organismal information, which was further embedded into a 300-dimensional feature vector. For the two continuous variables, pH and temperature, RBF [40, 41] was used to expand each continuous value $x$ into a feature vector $\boldsymbol{e}$ of dimension $M$, as follows:


(2) \begin{equation*} {e}_m(x)=\exp \left(-\gamma{\left\Vert x-{\mu}_m\right\Vert}^2\right) \end{equation*}

where $\gamma$ controls the shape of the radial kernel (we set $\gamma =10$).$\left\{{\mu}_m\right\}$ is a list of centers ranging from the minimum to the maximum of the corresponding features with a stride of 0.1. The ${e}_m(x)$ was further mapped linearly onto a 300-dimensional feature vector. Finally, the three 300-dimensional feature vectors were summed to obtain the auxiliary-term coding vectors.

### Multitask deep learning model construction

Considering that the enzyme catalysis efficiency requires a comprehensive evaluation of *k*_cat_ and *K*_m_, and there is some intrinsic connection between the two constants. The CGC multitask deep-learning model developed by Tencent contains expert modules specific to each task and shared expert modules between tasks, adopting a gating network to dynamically adjust the degree of contribution of the specific and shared modules to each task, achieving fast prediction of multiple tasks simultaneously, while reducing the seesaw phenomenon that may be caused by all shared expert modules [45]. The gating network can be flexibly adapted to different tasks, which can better control the information flow, reduce model parameters and computation, and improve the efficiency of model training and prediction. Therefore, we develop a multitask deep-learning framework based on the CGC framework to predict *k*_cat_ and *K*_m_ simultaneously and accurately. Referring to the CGC framework [45], we construct a multitask deep-learning model, containing two components: the expert layer and the tower layer.

#### Expert layer

The expert layer consists of the shared module (shared experts) and two expert modules (A and B). Dynamically adjusting the contribution of information from the expert and shared modules to each prediction task through a gating network [50]. The gating network is a single-layer feed-forward network using Softmax [51] as the activation function (Fig. 1). It acted as a selector to generate selection probabilities and computed the weighted sum of all selection vectors (i.e. the output of expert). The gated network for task $k$ is formalized as follows:


(3) \begin{equation*} {w}^k(x)=\mathrm{Softmax}\left({W}_g^kx\right) \end{equation*}

(4) \begin{equation*} {g}^k(x)={w}^k(x){S}^k(x) \end{equation*}

where $x$ is the input representation. ${W}_g^k\in{R}^{\left({m}_k+{m}_s\right)\times d}$ is a parameter matrix. ${m}_k$ and ${m}_s$ are the number of shared experts and task ${k}^{\prime }s$ specific experts, respectively. $d$ is the dimension of input representation. ${w}^k(x)$ is a weighting function to calculate the weight vector of task $k$ through the gating network. ${S}^k(x)$ is a selected matrix composed of all selected vectors including shared experts and task ${k}^{\prime }s$ specific experts:


(5) \begin{equation*} {S}^k(x)={\left[{E}_{\left(k,1\right)}^T,{E}_{\left(k,2\right)}^T,\dots, {E}_{\left(k,{m}_k\right)}^T,{E}_{\left(s,1\right)}^T,{E}_{\left(s,2\right)}^T,\dots, {E}_{\left(s,{m}_s\right)}^T\right]}^T \end{equation*}

#### Tower layer

The tower layer was used to output the two regression prediction tasks. Each tower contains three fully connected layers. Using the fusion representation ${g}^k(x)$ as the input, ${y}^k(x)$ as the prediction for task $k$:


(6) \begin{equation*} {y}^k(x)={t}^k\left({g}^k(x)\right) \end{equation*}

where ${t}^k$ denotes the tower network of task $k$. The activation function is ReLU [52] for the output. The output of each tower is a one-dimensional vector representing the predicted *k*_cat_ or *K*_m_ value.

#### Model parameter

Adam optimizer [53] and L2 loss were used to train the model. The training process aims to minimize the loss function. The main parameters of the CGC-based multitask model were set as follows: learning rate: 1𝑒−4; expert’s number: 4; expert’s layer: 1; expert’s vector dimension: 768; expert’s dropout rate: 0.2; batch size: 1; epochs: 50; tower’s layer: 3; tower’s vector dimensions: 128, 128, or 1. These different settings were explored based on the RMSE and *R*^2^ of validation datasets during the hyperparameter tuning to find which hyperparameter is better for improving the MPEK performance [13]. The learning rate, batch size, epochs, dropout rate, and expert’s number were mainly adjusted, and the detailed tuning process is shown in Table S2. MPEK was trained by a NVIDIA HGX A100 with 80G memory.

### Evaluation metrics

For regression tasks, PCC, *R*^2^, and the RMSE, which indicate the linear correlation, the fit degree of the model to the data, and the difference between the predicted and log10-transformed *k*_cat_ and *K*_m_ values, respectively, were used as evaluation metrics [54]. We used the HR to evaluate the case study predictions.


(7) \begin{equation*} PCC=\frac{\sum_{i=1}^n\left({y}_{ie}-{\overline{y}}_e\right)\left({y}_{ip}-{\overline{y}}_p\right)}{\sqrt{\sum_{i=1}^n{\left({y}_{ie}-{\overline{y}}_e\right)}^2}\sqrt{\sum_{i=1}^n{\left({y}_{ip}-{\overline{y}}_p\right)}^2}} \end{equation*}

(8) \begin{equation*} {R}^2=1-\frac{\sum_{i=1}^n{\left({y}_{ie}-{y}_{ip}\right)}^2}{\sum_{i=1}^n{\left({y}_{ie}-{\overline{y}}_e\right)}^2} \end{equation*}

(9) \begin{equation*} RMSE=\sqrt{\frac{1}{n}\sum_{i=1}^n{\left({y}_{ip}-{y}_{ie}\right)}^2} \end{equation*}

where${y}_{ie}$ is the experimental *k*_cat_ or *K*_m_ values. ${y}_{ip}$ is the predicted *k*_cat_ or *K*_m_ values. ${\overline{y}}_e$ is the average of the experimental *k*_cat_ or *K*_m_ values. ${\overline{y}}_p$ is the average of the predicted *k*_cat_ or *K*_m_ values. $n$ is the number of entries in the datasets (training dataset, validation dataset, and test dataset).


(10) \begin{equation*} HR=\frac{1}{N}{\sum}_{i=1}^N hit(i) \end{equation*}

where $N$ is the number of samples. $hit(i)$ whether the predicted *k*_cat_/*K*_m_ value for the $i$-th sample matches the trend of enzyme catalytic efficiency change. If yes, its value is 1, otherwise it is 0.

**Key Points**

- A multitask learning method, MPEK, was proposed to predict the values of *k*_cat_ and *K*_m_ simultaneously.
- Using pretrained language models to characterize the enzyme and substrate and integrating pH, temperature, and organism information to make the enzyme catalysis characterization more comprehensive.
- The proposed framework outperformed the existing state-of-the-art methods on the same test dataset.
- An online server was provided to predict *k*_cat_ and *K*_m_ values easily and efficiently.

## Supplementary Material

Supplementary_Materials_bbae387

## Data Availability

The MPEK web server is freely available at http://mathtc.nscc-tj.cn/mpek. The trained models and the MPEK standalone source code can be found at https://github.com/kotori-y/mpek. All relevant data supporting the results of this study are provided in Source data.
